# Incorporating Portfolio Uncertainty in Decision Rules for Healthcare Resource Allocation

**DOI:** 10.3390/healthcare9030325

**Published:** 2021-03-14

**Authors:** Pedram Sendi, Amiram Gafni, Stephen Birch, Stephen D. Walter

**Affiliations:** 1 Institute for Clinical Epidemiology, Basel University Hospital, CH-4031 Basel, Switzerland; 2 Centre for Health Economics and Policy Analysis, Department of Health Research Methods, Evaluation, and Impact, McMaster University, Hamilton, ON L8S4K1, Canada; gafni@mcmaster.ca; 3 Centre for the Business and Economics of Health, University of Queensland, St Lucia, QLD 4072, Australia; stephen.birch@uq.edu.au; 4 Manchester Centre for Health Economics, University of Manchester, Manchester M139PL, UK; 5 Department of Health Research Methods, Evaluation, and Impact, McMaster University, Hamilton, ON L8S4K1, Canada; walter@mcmaster.ca

**Keywords:** cost-effectiveness analysis, resource allocation, risk aversion, uncertainty, opportunity costs

## Abstract

Cost-effectiveness analysis is widely adopted as a means to inform policy and decision makers in setting priorities for healthcare resource allocation. In resource-constrained settings, decision makers are confronted with healthcare resource reallocation decisions, e.g., moving funds from one or more existing healthcare programs to fund new healthcare programs. The decision-making plane (DMP) has been developed as a means to graphically present the results of reallocating available healthcare resources when healthcare program costs and effects are uncertain. Mapping a value function over the DMP allows the analyst to value all possible combinations of net costs and net effects that may result from reallocating available healthcare resources under conditions of uncertainty. In this paper, we extend this approach to include a change in portfolio risk, stemming from a change in the portfolios of funded healthcare programs, as an additional source of uncertainty, and demonstrate how this can be incorporated into the value function over net costs and net effects for a risk-averse decision maker. The methodology presented in this paper is of particular interest to decision makers who are risk averse, as it will help to better incorporate their preferences in the process of deciding how to best allocate scarce healthcare resources.

## 1. Introduction

The resources allocated to healthcare are scarce; therefore, decision makers (DMs) must decide how best to allocate them. Resource scarcity occurs in different contexts, i.e., with fixed, shrinking or increasing budgets, and so DMs must decide where additional resources will come from to fund new healthcare programs [1,2,3]. In the case of increasing budgets, opportunities to forgo funding for competing (health or non-health) programs are implicit. If available resources are to be used efficiently, DMs must consider the opportunity cost (benefits forgone) of resources allocated to new healthcare programs, or what Williams (1983) described as ensuring *“… that the value of what is gained from an activity outweighs the value of what has to be sacrificed”* [4].

Cost-effectiveness analysis (CEA) is presented and used as a means to maximize the health benefits produced from available resources [5]. The analytical tool of CEA is the incremental cost-effectiveness ratio (ICER), which is compared to a threshold value (λ), corresponding to the shadow price of the budget constraint, to determine whether a new program represents an improvement in efficiency in the use of available resources compared to the existing allocation of the same resources [6,7]. Using a threshold value as a cut-off point for identifying efficient programs assumes constant returns to scale and the perfect divisibility of programs [1,2,8]. Birch and Gafni [1] developed an alternative decision rule, which relaxes these assumptions and avoids use of a threshold value. The alternative rule identifies the additional benefits and resource requirements for a new program and directly compares the additional benefits with the benefits forgone from meeting the additional resource requirements by reallocating resources from other programs. In doing so, unlike the threshold ICER value approach, it satisfies the efficiency requirement presented by Williams [4]. In the present paper, we do not elaborate on the debate about the decision rules of cost-effectiveness analysis and assume that the reader is familiar with it by referring to the respective literature [1,2,3,8].

While this alternative decision rule is straightforward in the absence of uncertainty associated with program costs and effects, in reality, both costs and effects are stochastic. The decision-making plane (DMP) graphically presents the distribution of all potential net cost and net effect pairs that may result from reallocating resources between programs under uncertainty [9]. Furthermore, a value function that describes the value of each combination of net cost and net effect may be mapped over the DMP to extend the probabilistic interpretation of the DMP with the DM’s preferences over uncertain costs and effects [10]. However, a risk-averse DM may also be interested in knowing whether resource allocation decisions are variance increasing or variance decreasing and the extent of these differences in the variance, assuming less variance is preferred, ceteris paribus. To this end, DMs may be risk averse towards costs, as they may need to meet resource constraints [11,12,13] and may be reluctant to exceed the available resources. In addition, DMs may be risk averse towards health outcomes since health is not a transferable good, i.e., those whose health is increased by funded healthcare programs cannot compensate individuals whose health is reduced by discontinuing other programs as a result of reallocating resources [12,14,15]. Changes in program portfolio variance therefore represent an additional dimension of uncertainty not captured by the joint distribution presented on the DMP, and need to be considered in resource reallocation decisions. Different resource allocation scenarios may lead to the same or similar distributions of net costs and effects on the DMP. In order to capture and value the potential change in the variance of the portfolio of funded programs, which is important to inform risk-averse decision makers, we therefore need to extend the current approach for valuing uncertain outcomes on the DMP [10].

The present paper is different from the current stream of literature addressing risk aversion in cost-effectiveness analysis [16,17,18] because it explicitly considers risk aversion in healthcare resource reallocation decisions. As explained above, instead of using a threshold cost-effectiveness ratio as a decision rule, which assumes constant returns to scale, complete divisibility of programs, and that an additional stream of resources is available at a constant marginal opportunity cost, we relax these assumptions and address risk aversion when the portfolio of the funded program is modified (i.e., taking into account the opportunity cost). This paper is therefore relevant to DMs faced with healthcare resource constraints when aiming to maximize the health of the population, while, at the same time, addressing the uncertainty associated with costs and effects of healthcare programs, assuming a risk-averse decision maker. This may be of the utmost relevance in settings where healthcare resource constraints limit extensive healthcare program coverage, and health authorities therefore need to decide which healthcare programs to fund (and which programs to forgo).

In the Methods section, we review the alternative decision rule under certainty and uncertainty. We then introduce the idea of increasing or decreasing the program portfolio variance in costs and effects associated with reallocating resources. In the Results section, we show how a change in the program portfolio variance in costs and effects can be incorporated in and how this affects the value function with regard to net costs and net effects. Finally, we discuss areas for possible future research.

## 2. Methods

### 2.1. The Case of Certainty

The rationale behind the alternative decision rule is described in detail elsewhere [1,2,8,19]. Here, we review the formal definitions of the decision rule under certainty [9]. For simplicity, we refer to a fixed budget where only one program is cancelled to free up the additional resources required to fund a new program.

To fund *program A*, which will replace *program a*, we need to find a *program B*, which will be replaced by *program b*, such that both (1) and (2) hold:

ΔC(A) ≤ ΔC(B)(1)

and

ΔE(A) > ΔE(B)(2)

where ΔC(A) = C_A_ − C_a_ and ΔC(B) = C_B_ − C_b_, and ΔE(A) = E_A_ − E_a_ and ΔE(B) = E_B_ − E_b_.

Consider the example of four healthcare programs, as shown in Table 1. We would like to introduce new *program A*, which requires an additional USD 60,000,000 compared to the current *program a*. *Program A* generates 60 Life Years (LYs) more than *program a* for the same patient group. Assume that we identify *programs B* and *b*, which meet the requirements of Equations (1) and (2). *Program B*, if cancelled and replaced by *b*, releases USD 70,000,000, but reduces health outcomes by 20 LYs. Therefore, removing *program B* from the program portfolio to fund the additional costs of *program A* leads to a net gain of 40 LYs without the need to increase resources. As a hypothetical example, *program A* may reflect treating patients with lung cancer with surgery and radiochemotherapy, while *program a* may reflect treating the same patients with radiochemotherapy only. Moreover, as a hypothetical example, *program B* may reflect treating patients with Hepatitis C infection with antiviral drugs, while *program b* may represent not treating the same patients with antiviral drugs.

**Figure 1:**
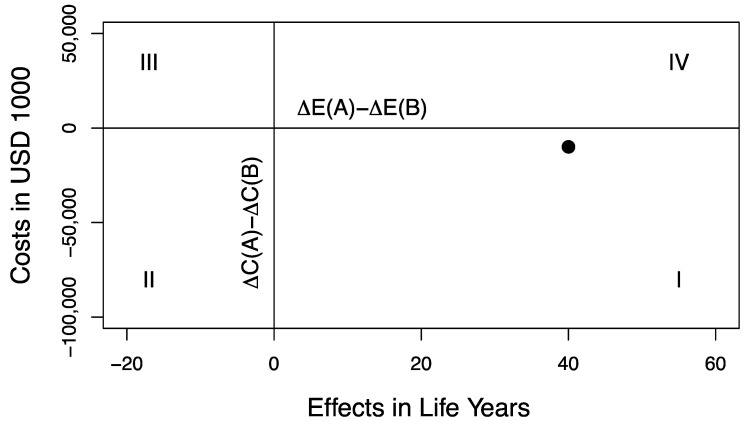
Decision-making plane. The dot in Quadrant I represents a healthcare resource reallocation policy that leads to a reduction of USD 10,000,000 in net costs and a net increase of 40 Life Years (see Table 1). This therefore reflects an unambiguous improvement in the allocation of scarce healthcare resources.

The results of this deterministic resource reallocation policy are displayed on the DMP (Figure 1). The DMP is divided into four quadrants by axes representing the net costs and net effects after resource reallocation. The requirements for efficient resource use of the alternative decision rule fall in Quadrant I (Figure 1). For the remainder of this paper, we refer to net costs and net effects to denote the costs and effects after resource reallocation, which are displayed on the DMP. We refer to net costs and effects as opposed to incremental costs and effects since more than two (mutually exclusive) programs are involved. We refer to program costs and effects to denote the costs and effects of the individual programs, i.e., *A*, *a*, *B* and *b*. Finally, we refer to portfolio costs and effects to denote the costs and effects of the jointly funded programs before and after reallocating resources, i.e., *Pf(A + b)* and *Pf(a + B)*, respectively.

### 2.2. The Case of Uncertainty

Because the costs and effects of healthcare programs are uncertain, the alternative decision rule may lead to a non-zero probability that reallocating available resources from *program B* to *program A* may lead to net costs and net effects that do not fall within Quadrant I of the DMP [9,20,21]. Consider the four programs shown in Table 2. The expected program costs and effects are identical to those used in the deterministic analysis (Table 1). We assume a normal distribution for program costs and program effects (following the central limit theorem, mean costs and mean effects are normally distributed if the sample size is sufficiently large, irrespective of the underlying distributions) and that costs and effects in each program are correlated with ρ = 0.5, indicating that increased health outcomes are associated with higher costs.

**Figure 2:**
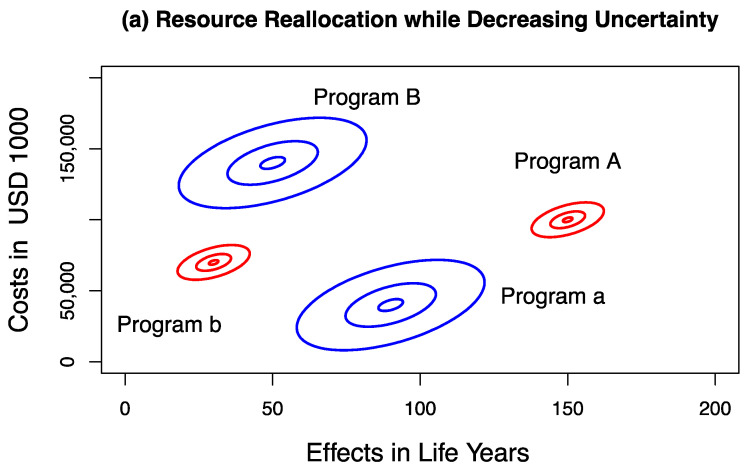

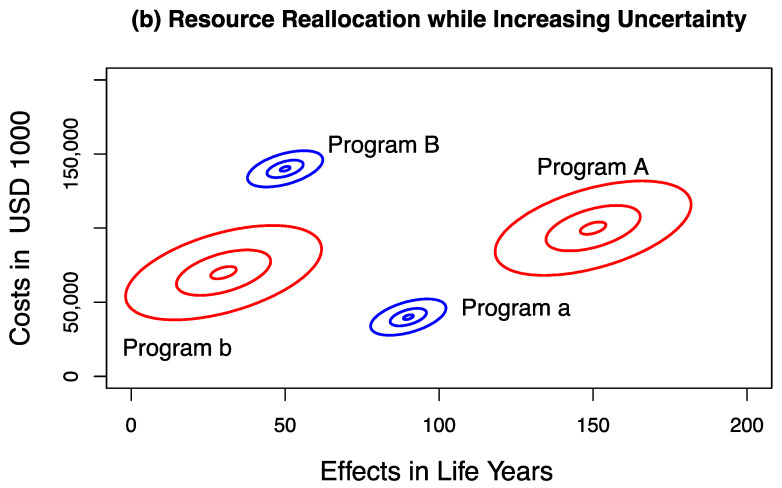
Healthcare resource reallocation while (**a**) decreasing and (**b**) increasing portfolio program uncertainty. The 5%, 50% and 95% credible ellipses for each program are drawn. The portfolio of jointly funded programs is indicated in red and blue colors. *Program A* and *program b* (both in red) represent the portfolio of funded programs after resource reallocation, and *program B* and *program a* (both in blue) represent the portfolio of funded programs before resource reallocation. By reallocating healthcare resources, *program a* is replaced by *program A*, and *program B* is replaced by *program b.* See also Scenario 1 and Scenario 2 in Table 2.

**Figure 3:**
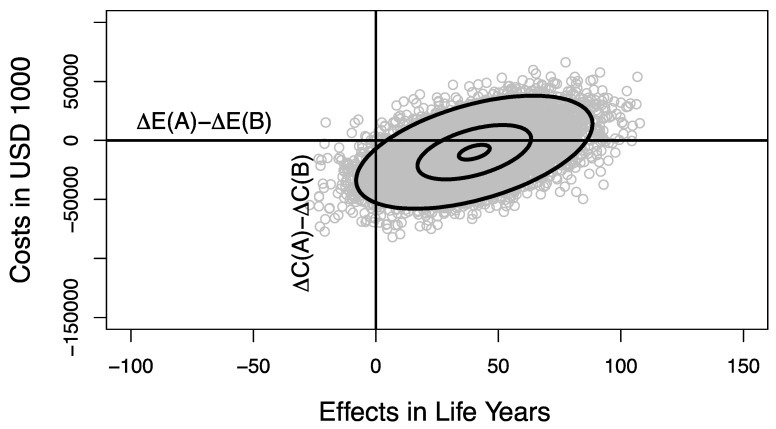
Joint distribution of net costs and net effects on the decision-making plane (DMP). The distribution of net costs and effects on the DMP were estimated by sampling 10,000 times from the four distributions defined in Table 2. Both Scenario 1 and Scenario 2 lead to the same distribution on the DMP. The 5%, 50% and 95% credible ellipses are drawn.

We present examples of resource allocation decisions that are variance decreasing or variance increasing. In the first example, we assume that new *program A* has a lower standard deviation for both costs and effects than existing *program a*, indicating that uncertainties in costs and uncertainties in effects are reduced by replacing *program a* with *program A* (Table 2, Scenario 1). We further assume that, by removing existing *program B* from the program portfolio, and providing *program b* instead, we further reduce the uncertainty, i.e., *program b* has a lower standard deviation for both costs and effects than *program B* (Table 2, Scenario 1 and Figure 2a). By reallocating resources between programs, we replace *portfolio Pf(a + B)* with *portfolio Pf(A + b)* (Figure 2a). The joint distribution of net costs and net effects on the DMP is shown in Figure 3 as a result of sampling 10,000 times from the four distributions defined in Table 2 and Figure 2a (Scenario 1) and estimating the net cost and net effect pairs for each sample using R (Version 3.6.1 and RStudio Version 1.2.1335, www.r-project.org, accessed February 28, 2021). Figure 3 shows the credible ellipses of the joint distribution of net costs and net effects that contain 95%, 50% and 5% of the samples, respectively, and illustrates the density of the joint distribution of net costs and net effects on the DMP. The probability that this reallocation policy will result in an unambiguous improvement in the efficiency of resource use is 67.7%, corresponding to the proportion of samples falling in Quadrant I of the DMP. There is a 2.0% probability that cancelling *program B* and replacing it with *program b* to release the additional resources required by *program A* will result in a net health loss (Quadrant II and III of the DMP) and a 30.3% probability that the available resources will be insufficient to support this reallocation, but will result in a net health gain (Quadrant IV of the DMP, Figure 3).

The bivariate normal distribution (BVN) of net costs and net effects on the DMP reflects the uncertainty associated with program costs and program effects of all four programs, i.e., those that are replaced and those that are supported by available resources. As such, the variances of the normal distributions displayed on the DMP are defined, in general, as

(3) $$\sigma_{C_{DMP}}^{2}=\sigma_{C_{A}}^{2}+\sigma_{C_{a}}^{2}+\sigma_{C_{B}}^{2}+\sigma_{C_{b}}^{2}\;\mathrm{for}\;\mathrm{costs}\;\mathrm{and}$$

(4) $$\sigma_{E_{DMP}}^{2}=\sigma_{E_{A}}^{2}+\sigma_{E_{a}}^{2}+\sigma_{E_{B}}^{2}+\sigma_{E_{b}}^{2}\;\mathrm{for}\;\mathrm{effects}\;$$

where $\sigma_{C_{DMP}}^{2}$ and $\sigma_{E_{DMP}}^{2}$ denote the variances of net costs and net effects, respectively. By using the joint distribution of net costs and net effects on the DMP for decisions about whether to reallocate resources to *program A*, we cannot determine whether the uncertainty of the (new) program *portfolio Pf(A + b)* has been increased or reduced compared to the (old) *portfolio Pf(a + B)* or by how much. This is because a wide variety of possible combinations of variances for program costs and effects may lead to the same $\sigma_{C_{DMP}}^{2}$ and $\sigma_{E_{DMP}}^{2}$. Some of them may increase portfolio variance and some of them may reduce portfolio variance. However, this information is important for a risk-averse decision maker (i.e., one who prefers less risk in program portfolios, ceteris paribus). Because a portfolio with *program B* and *program a* has a larger variance than a portfolio with *program A* and *program b* (Figure 2a and Table 2, Scenario 1), reallocating available resources to *program A* and *b* is variance reducing.

Different healthcare programs may exhibit different levels of uncertainty. For example, antiretroviral treatment for HIV infection has been found to be variance increasing for both costs and effects [22]. Before potent antiretroviral treatment was available, patients with HIV infection had a very short life expectancy and healthcare costs were mainly accrued for the treatment of AIDS. With the advent of potent antiretroviral drugs, HIV-infected patients have a close to normal life expectancy and may die of competing risks, such as cancer or heart attacks. Therefore, the variance of program effects and program costs is increased. In contrast, Hepatitis B virus infection may involve patients developing liver cancer, which needs to be treated with expensive procedures [23]. A Hepatitis B vaccination is associated with a negligible disutility (pain, suffering, or side effects) and may prevent the follow-up costs of Hepatitis treatment. Therefore, Hepatitis B vaccination may reduce the variance of both program costs and program effects. 

Consider the same four healthcare programs described above, but with reversed uncertainties, as shown in Table 2 and Figure 2b (Scenario 2). New *program A* and *program b* now have a higher standard deviation for costs and effects than *program B* and *program a*. Therefore, by allocating available resources to *program A* and *b*, the overall uncertainty of the *portfolio Pf(A + b)* is increased compared to *Pf(a + B)* (Figure 2b and Table 2, Scenario 2). Since the variances of the joint distribution of net costs $\sigma_{C_{DMP}}^{2}$ and net effects $\sigma_{E_{DMP}}^{2}$ on the DMP reflect the sum of the variances of all four programs (see Equations (3) and (4)), the joint distribution on the DMP is identical for both Scenario 1 and Scenario 2 (Figure 3). Although both the variance-decreasing Scenario 1 (Table 2 and Figure 2a) and the variance-increasing Scenario 2 (Table 2 and Figure 2b) lead to the same joint distribution of net costs and net effects on the DMP (Figure 3), a risk-averse DM prefers Scenario 1 over Scenario 2, ceteris paribus. 

We therefore need to provide information about the uncertainty of the portfolios of programs to a risk-averse DM before and after resources are reallocated from *a* and *B* to *A* and *b* [21]. Resource reallocation to *A* and *b* would be preferred if

(5) $$\sigma_{C_{A}}^{2}+\sigma_{C_{b}}^{2}<\sigma_{C_{a}}^{2}+\sigma_{C_{B}}^{2}\;$$

ceteris paribus, or

(6) $$\sigma_{E_{A}}^{2}+\sigma_{E_{b}}^{2}<\sigma_{E_{a}}^{2}+\sigma_{E_{B}}^{2}\;\mathrm{ceteris}\;\mathrm{paribus}$$

Although there may be different approaches to presenting and valuing changes with regard to portfolio risk, in this paper, we present a simple but robust approach using the quartile coefficient of dispersion (QCD), which is less sensitive to skewness [24]. The QCD uses the first (*Q*_1_) and third (*Q*_3_) quartiles of a distribution for the costs or effects of the portfolio of funded programs and is estimated as

(7) $$QCD=\frac{Q_{3}-Q_{1}}{Q_{3}+Q_{1}}$$

A higher QCD indicates greater dispersion, and can thus be used to compare the degree of uncertainty between programs descriptively. There are other metrics that may be used to describe dispersion, such as the coefficient of variation, commonly used in finance, which is estimated by taking the ratio of the standard deviation to the mean. However, in a highly skewed distribution, the standard deviation is strongly affected by the tail of the distribution and outliers. The QCD is less sensitive to skewness as quartiles of a distribution are used for estimating the QCD, and differences between the extreme values in the tail are disregarded. 

We can now evaluate the change in uncertainty by comparing the QCDs for the costs and effects of *Pf(A + b)* and *Pf(a + B*). Consider Scenario 1 (Table 2), where portfolio uncertainty is reduced. The QCD of portfolio *Pf(a + B)* for portfolio costs and portfolio effects before implementing program A is 0.069 and 0.089, respectively (Table 3). After available resources have been reallocated to *A* and *b*, the QCD for portfolio costs and portfolio effects is reduced to 0.028 and 0.026, respectively (Table 3). On the other hand, if we consider Scenario 2 (Table 2), where portfolio uncertainty is increased, the QCD of portfolio *Pf(a + B*) for costs and effects, before reallocating available resources to *A* and *b*, is 0.027 and 0.034, respectively (Table 3). After reallocating resources to *A* and *b*, the QCD of portfolio *Pf(A + b)* for costs and effects is increased to 0.074 and 0.070, respectively (Table 3). As such, the alternative decision rule increases the portfolio risk with regard to costs and effects by factors of 2.7 and 2.1, respectively. Including change in portfolio variance, as described by a change in (for example) QCD, in the valuation of outcomes on the DMP is therefore important in order to inform risk-averse decision makers. This will be further discussed in the section “Valuing Outcomes on the Decision-Making Plane”. 

The probabilities of net costs and net effects falling in each quadrant of the DMP plane as a result of reallocating resources may also be calculated analytically. In situations where a disease model can be evaluated analytically, this would be the preferred approach to describe the structure of the system. However, in more complicated disease models, analysts usually resort to simulation for evaluating a cost-effectiveness model. As shown in Figure 3, the joint distribution of net costs (*C_DMP_*) and net effects (*E_DMP_*) on the DMP follows a BVN distribution, and the quadrant probabilities can be derived by integration of the BVN. 

For computational purposes, it is convenient to standardize the variables on the DMP, as follows. The x- and y-axes in the resultant standardized BVN are:

$$x=\frac{E_{DMP}-\mu_{E_{DMP}}}{\sigma_{E_{DMP}}}\;\mathrm{and}\;y=\frac{C_{DMP}-\mu_{C_{DMP}}}{\sigma_{C_{DMP}}}$$

where $\mu_{E_{DMP}}$ and $\sigma_{E_{DMP}}$ are the mean and standard deviation of the observed values of $E_{DMP}$, and $\mu_{C_{DMP}}$ and $\sigma_{C_{DMP}}$ are the corresponding parameters for $C_{DMP}$.

In order to calculate the probability $p_{1}$ for Quadrant I, for instance, we can use the bivariate integral

(8) $$p_{1}=\int_{-\infty }^{0}\int_{0}^{\infty }\varphi \left(x,y,\rho \right)dx.dy$$

where φ(x,y,ρ) is the probability distribution function for the standardized BVN distribution, with a correlation ρ between the variables *x* and *y*.

The probabilities for the other three quadrants are as follows:

(9) $$p_{2}=\;\int_{-\infty }^{0}\int_{-\infty }^{0}\varphi \left(x,y\right)dx.dy\;\mathrm{for}\;\mathrm{Quadrant}\;\mathrm{II}$$

(10) $$p_{3}=\;\int_{0}^{\infty }\int_{-\infty }^{0}\varphi \left(x,y\right)dx.dy\;\mathrm{for}\;\mathrm{Quadrant}\;\mathrm{III}$$

(11) $$p_{4}=\int_{0}^{\infty }\int_{0}^{\infty }\varphi \left(x,y\right)dx.dy\;\mathrm{for}\;\mathrm{Quadrant}\;\mathrm{IV}$$

In order to implement these calculations, one requires the input values for the parameters $\mu_{E_{DMP}}$, $\sigma_{E_{DMP}}$, $\mu_{C_{DMP}}$ and $\sigma_{C_{DMP}}$, together with the correlation ρ between $E_{DMP}$ and $C_{DMP}$. The means in the two dimensions of the DMP plane are

$$\mu_{E_{DMP}}=\Delta E\left(A\right)-\Delta E\left(B\right)$$

and

$$\mu_{C_{DMP}}=\Delta C\left(A\right)-\Delta C\left(B\right)$$

with appropriate values of the component costs and effects of each program being used to estimate the numerical coordinates of the center of the ellipses in Figure 3. The variances of the ellipses’ *x* and *y* distributions are given by Equations (3) and (4). 

The correlation required in (8)–(11) can be derived as:

$$\rho =\frac{\rho_{A}\cdot \sigma_{C_{A}}\cdot \sigma_{E_{A}}+\rho_{a}\cdot \sigma_{C_{a}}\cdot \sigma_{E_{a}}+\rho_{B}\cdot \sigma_{C_{B}}\cdot \sigma_{E_{B}}+\rho_{b}\cdot \sigma_{C_{b}}\cdot \sigma_{E_{b}}}{\sigma_{E_{DMP}}\cdot \sigma_{C_{DMP}}}$$

where $\rho_{A}$ is the correlation between the cost and effect values within *program A* and, similarly, for *programs a, B* and *b*. 

Numerical evaluation of the integrals, such as in (8), will provide accurate values for the quadrant probabilities of the joint distribution displayed in Figure 3, and hence provide a means to validate the simulation results [25]. However, these evaluations (both from the integrals and from the simulations) are accurate only if the inherent assumptions are correct. In particular, the values of the input parameters must be correct, and the data should be normally distributed. Non-trivial departures from these assumptions will lead to inaccurate results from both the analytic solution and from simulated samples.

A related issue here is that the parameters may need to be estimated from sample data and, therefore, unless sample sizes are very large, there will be sample variation that affects the parameter estimates and, hence, the estimated quadrant probabilities. As such, there will be additional uncertainty in the positions and sizes of the ellipses in Figure 3, and the estimated quadrant probabilities will be affected; the ellipses will become more inflated, with consequent changes in their component quadrant probabilities. It may be possible to extend our analytic approach to take into account the sample variation in the estimated parameters, but this is something that is beyond the scope of the current paper.

## 3. Results

### Valuing Outcomes on the Decision-Making Plane

Decision makers may not give equal value to every potential outcome of a reallocation of available resources on the DMP. Gafni et al. [10] suggested mapping a value function over the DMP that reflects a DM’s preferences for all possible net cost and net effect pairs using loss and gain functions. Such value functions imply that not every resource reallocation outcome in Quadrant I of the DMP may be considered as equally good and not every resource reallocation outcome outside Quadrant I (i.e., Quadrants II, III and IV) as equally bad [10]. Gafni et al. suggested, as a hypothetical example, that loss *f* and gain *g* functions for each quadrant of the DMP may be defined as shown in Table 4, where x = ΔE(A) − ΔE(B) and y = ΔC(A) − ΔC(B) [10]. The exponents α_1_ and α_2_ represent the decision maker’s valuation of resource reallocation outcomes x and y and reflect a progressively increasing (if >1) or decreasing (if <1) rate of gain or loss as one moves away from the origin of the DMP [10]. The exponents α_1_ and α_2_ are specific to a DM (here we will assume α_1_ = α_2_ = 2), and can be interpreted as a risk aversion parameter [10]. 

In the present paper, we introduce further exponents (*β*_1_ and *β*_2_; Table 4), which are additional parameters used to modify a risk-averse decision maker’s valuation of resource reallocation outcomes depending on whether a reallocation is variance increasing or variance decreasing, and the extent of these differences in the variance. The value of gains (and losses) in the different quadrants of the DMP may be reduced (or increased) if reallocating resources is variance increasing (i.e., *β*_1_ < 1 and *β*_2_ > 1), since this may represent a disutility to a risk-averse DM. On the other hand, if moving from *portfolio Pf(a + B)* to *portfolio Pf(A + b)* is variance decreasing, and hence represents an increase in utility to the DM, then the value of net gains (and net losses) on the DMP may be increased (or decreased) (i.e., *β*_1_ > 1 and *β*_2_ < 1). If reallocating available resources between programs is neither variance increasing nor variance decreasing, then *β*_1_ = *β*_2_ = 1. Using the net gain and loss functions defined above (Table 4), we can estimate the value of total expected net gain and total expected net loss of reallocating available resources in the two scenarios, assuming, for demonstration purposes, *β*_1_ = 1.1 and *β*_2_ = 0.9 for portfolio risk reduction and *β*_1_ = 0.9 and *β*_2_ = 1.1 for an increase in portfolio risk (Table 4 and Table 5). Depending on the DM’s value function and whether the uncertainty of both costs and effects are increased or decreased in the same or opposite directions, one could define a separate correcting factor for costs *β_*1*C_* and effects *β_*1*E_* for gains (and β_2C_ and β_2E_ for losses). However, for demonstration purposes, we assume *β_*1*C_* = *β_*1*E_* = *β*_1_. We emphasize that these are hypothetical estimates and are presented to demonstrate how a change in portfolio variance may affect the value function. In reality, one would need to elicit the respective preferences from the DM (i.e., his/her value function). 

To estimate expected net gain and expected net loss using the value function defined above (Table 4), we drew 10,000 samples from the original distributions for program costs and program effects, as shown in Table 2 and rescaled costs (Costs/USD 10,000,000, i.e., units of USD 10 M) and effects (LY/10, i.e., units of 10 LY) to facilitate a comparison of costs and effects. This ensures that costs and effects contribute equally to the value function; otherwise, the impact of health outcomes on net gains and net losses would be negligible and net gains and net losses would primarily reflect the valuation of costs. This does not affect the quadrant probabilities, nor does it affect the relative ranking of net cost–net effect pairs on the DMP. Note that the rescaling “creates”, in our example, a single and constant “currency”, independent of the magnitude of the exponents used in the value function for costs and effects. For each quadrant on the DMP, the expected net gain and net loss were computed by multiplying the expected values within a quadrant by the percentage of samples falling in that quadrant. As shown in Table 5, assuming *β_1_ = β*_2_ *=* 1, the net gain and net loss are the same for both the variance-decreasing scenario and the variance-increasing scenario, with the small difference in net gain and net loss representing sampling variations. If the reallocation of available resources on the DMP is penalized (*β*_1_ = 0.9, *β*_2_ = 1.1) due to an increase in uncertainty for both costs and effects (Scenario 2, Table 5), which may represent a disutility to the decision maker, then the net gain is reduced to 17.50. On the other hand, if available resource reallocation leads to a variance reduction (Scenario 1, Table 5), the decision maker may perceive a higher utility (*β*_1_ = 1.1, *β*_2_ = 0.9) and the net gain is then increased to 33.04. In our example, net losses are reduced to 0.69 for the variance-reducing scenario and increased to 1.03 for the variance-increasing scenario. That is, the ranking of the reallocation decision changes from being indifferent between Scenario 1 and Scenario 2, when ignoring the change in portfolio risk, to preferring Scenario 1 over Scenario 2 when we include the change in portfolio risk in the value function for a risk-averse decision maker.

## 4. Discussion

In this paper, we extended the previous work dealing with the uncertainty of the net costs and net effects when reallocating resources to include the risk aversion of the DM towards an increase in portfolio risk. In the present paper, we take a different approach than other current studies, which address risk aversion and affordability within the context of cost-effectiveness analysis. In a recent paper, for example, Lomas suggested a modification of the net benefit approach using a threshold ratio as a decision rule, which is adjusted depending on the budget impact of a new program [26]. However, once an adjusted threshold ratio has been defined, it assumes the perfect divisibility of programs and constant marginal opportunity costs. Our approach directly compares the benefits gained by a new program after a candidate program (or a set of programs) has been removed, i.e., no assumptions about the divisibility of programs and marginal opportunity costs are made. A different approach in the presence of uncertain costs and effects, and in the absence of any information on candidate programs to be deleted, would be to directly address the budget impact using the cost-effectiveness affordability curve [27]. This approach, however, assumes that an additional stream of resources is available, where the opportunity costs may be non-health related. 

How changes in portfolio risk translate into a value function depends on the preferences of a decision maker. In this paper, we assume that a DM prefers less risk, and this is reflected in a smaller variance, ceteris paribus. Other value functions, such as a preference for min–max reduction, would also be possible. We would then seek to reduce the spread of the costs and effects of the portfolio after resource reallocation. A mean–variance utility function may also be appropriate to model the trade-off between expected return and risk [11,28,29]. Incorporating changes in portfolio risk into a value function is an empirical matter. In the present paper, we reduced (or increased) the net gain (or net loss) on the DMP when introducing a new program that is variance increasing. This implies that the exponent of the value function for net gains and net losses is modified. Recent empirical research has focused on evaluating the value function of a sample of the general public in France for different resource reallocation policies [30,31]. Change in portfolio risk, however, is an additional level of uncertainty that may impact the decision maker’s valuation of net costs and net effects. Linking the change in portfolio risk to the valuation of net costs and effects requires a preference function, such as a utility function or prospect theory value function, which is subject to future research and beyond the scope of the present paper.

In the present paper, we assumed risk aversion towards both costs and effects. However, this approach could be extended to only include risk aversion towards effects and risk neutrality towards costs (or vice versa). In that case, we would limit the value function for gains and losses to health effects only and consider a resource reallocation as long as expected costs do not exceed the anticipated budget level. Furthermore, the value functions used for estimating gains and losses could be modified depending on the preferences of a decision maker, e.g., if a decision maker would prefer to weigh uncertain gains in health outcomes more than potential losses, the exponents of the value function could be adjusted accordingly. 

## 5. Conclusions

Changes in the before–after variance of a portfolio of healthcare programs may represent important components of portfolio risk that need to be assessed and included in the value function of net gains and net losses over the DMP. Empirical research is needed to evaluate how DMs value such changes in portfolio risk. In addition, future research should focus on defining and linking a preference function, such as a utility function or prospect theory value function, to the change in the variance of the costs and effects of the portfolio of funded programs. The methodology presented in this paper is of particular interest to DMs who are risk averse, as it will help to better incorporate their preferences in the process of deciding how to best allocate scarce healthcare resources.

## Figures and Tables

**Table 1:** Costs and effects of four hypothetical programs for resource reallocation.

Programs	Costs (C) (In USD 1000)	Effects (E) (In Life Years)	ΔC (In USD 1000)	ΔE (In Life Years)
Program A	100,000	150	60,000	60
Program a	40,000	90		
Program B	140,000	50	70,000	20
Program b	70,000	30		

Resource reallocation policy, where *program a* is replaced with *program A*, and *program B* is replaced with *program b*. This resource reallocation policy leads to a net gain of USD 10,000,000 and 40 Life Years (see Figure 1). ΔC denotes incremental costs; ΔE denotes incremental effects.

**Table 2:** Costs and effects of four programs for resource reallocation with decreasing (Scenario 1) and increasing (Scenario 2) variance.

Scenario	Programs	Mean Costs (In USD 1000)	SD of Costs (In USD 1000)	Mean Effects (In Life Years)	SD of Effects (In Life Years)
Sc. 1	Program A	100,000	5000	150	5
	Program a	40,000	13,000	90	13
	Program B	140,000	13,000	50	13
	Program b	70,000	5000	30	5
Sc. 2	Program A	100,000	13,000	150	13
	Program a	40,000	5000	90	5
	Program B	140,000	5000	50	5
	Program b	70,000	13,000	30	13

Independence of costs and effects between programs is assumed. For a graphical representation of the four programs in each scenario, see Figure 2a (Scenario 1) and Figure 2b (Scenario 2).

**Table 3:** Quartile coefficient of dispersion for costs and effects in two scenarios (using 10,000 samples) for resource reallocation (variance-decreasing Scenario 1 and variance-increasing Scenario 2).

Portfolio Costs and Effects	Variance-Decreasing Scenario 1	Variance-Increasing Scenario 2
Costs (A + b)	0.028	0.074
Effects (A + b)	0.026	0.070
Costs (a + B)	0.069	0.027
Effects (a + B)	0.089	0.034

**Table 4:** Loss and gain function for outcomes in the four quadrants of the DMP.

Quadrant	Loss	Gain
SE (I)	*f_SE_ =* 0	*g_SE_*= |y|^α2β1^ + |x|^α1β1^
SW (II)	*f_SW_* = |x|^α1β2^	*g_SE_* = |y|^α2β1^
NW (III)	*f_NW_=* |y|^α2β2^ + |x|^α1β2^	*g_NW_* = 0
NE (IV)	*f_NE_ =* |y|^α2β2^	*g_NE_ =* |x|^α1β1^

X = ΔE(A) − ΔE(B); y = ΔC(A) − ΔC(B); α_1_ = α_2_ = 2 (represents the decision maker’s value function without adjustment for change in portfolio variance); β_1_ and β_2_ are factors that adjust the decision maker’s value function for a change in portfolio variance.

**Table 5:** Net gain and net loss by reallocating resources for two scenarios (using 10,000 samples) for resource reallocation (variance-decreasing Scenario 1 and variance-increasing Scenario 2).

Exponent	Scenario 1 Decreasing Uncertainty		Scenario 2 Increasing Uncertainty	
	Net Gain	Net Loss	Net Gain	Net Loss
*β*_1_ = *β*_2_ = 1	23.99	0.82	23.96	0.84
*β*_1_ = 0.9, *β*_2_ = 1.1			17.50	1.03
*β*_1_ = 1.1, *β*_2_ = 0.9	33.04	0.69		

Costs (C) and effects (E) were rescaled by C/USD 10 M and E/10 LY.

## Data Availability

Available on request from the authors.
